# Sources of Variation in Assessing Canopy Reflectance of Processing Tomato by Means of Multispectral Radiometry

**DOI:** 10.3390/s19214730

**Published:** 2019-10-31

**Authors:** Giorgio Gianquinto, Francesco Orsini, Giuseppina Pennisi, Stefano Bona

**Affiliations:** 1 Department of Agricultural and Food Sciences (DISTAL), University of Bologna, Viale Fanin, 44, 40127 Bologna, Italy; giorgio.gianquinto@unibo.it (G.G.); f.orsini@unibo.it (F.O.); 2 Department of Agronomy, Food, Natural resources, Animals and Environment (DAFNAE), University of Padova, Viale dell’Università, 16, 35020 Legnaro (PD), Italy; stefano.bona@unipd.it

**Keywords:** passive sensor, sampling methodology, bootstrap analysis, vegetation indices, green ratio of vegetation index, NIR/560 or GVI (Green Vegetation Index), green normalized difference vegetation index, normalized difference vegetation index on greenness (G-NDVI), soil mulching, solanum lycopersicum L

## Abstract

Canopy reflectance sensors are a viable technology to optimize the fertilization management of crops. In this research, canopy reflectance was measured through a passive sensor to evaluate the effects of either crop features (N fertilization, soil mulching, appearance of red fruits, and cultivars) or sampling methods (sampling size, sensor position, and hour of sampling) on the reliability of vegetation indices (VIs). Sixteen VIs were derived, including seven simple wavelength reflectance ratios (NIR/R460, NIR/R510, NIR/R560, NIR/R610, NIR/R660, NIR/R710, NIR/R760), seven normalized indices (NDVI, G-NDVI, MCARISAVI, OSAVI, TSAVI, TCARI), and two combined indices (TCARI/OSAVI; MCARI/OSAVI). NIR/560 and G-NDVI (Normalized Difference Vegetation Index on Greenness) were the most reliable in discriminating among fertilization rates, with results unaffected by the appearance of maturing fruits, and the most stable in response to different cultivars. Black mulching film did not affect NIR/560 and G-NDVI behavior at the beginning of the growing season, when the crop is more responsive to N management. Due to a moderate variability of NIR/560 and G-NDVI, a small sample size (5–10 observations) is sufficient to obtain reliable measurements. Performing the measurements at 11:00 and 14:00 and maintaining a greater distance (1.8 m) between plants and instrument enhanced measurement consistency. Accordingly, NIR/560 and G-NDVI resulted in the most reliable VIs.

## 1. Introduction

In recent years, interest in multispectral radiometry in crop production has increased, having been successfully investigated in fertilization and irrigation management [1,2,3,4,5,6], and disease and pest control [7,8,9]. Optical sensors have proven to be particularly useful in maximizing N use efficiency and minimizing N losses. Among proposed models, image processing based on aerial photography considers a field-scale observation, whereas plot-scale is achieved when using portable multispectral radiometers, and leaf-scale is provided by portable chlorophyll and/or flavonols meters [2,5,10,11,12,13,14,15]. As plant/canopy observation is closer to the crop, predictions of chlorophyll and N content in plant tissues are more accurate [2], but less consideration is given to variability between plants and leaves, which translates into the need of larger sample numbers. On the other hand, the application of aerial crop remote sensing within precision agriculture in Europe is still scarcely adopted [2]. It is probably not due to a lack of interest from farmers but due to commercial systems being too expensive compared to the relatively low economic gains. Accordingly, the adoption of portable canopy reflectance sensors may represent a cheaper and appealing technology to optimize the fertilization management of crops. Their use has been fruitfully adopted in cereals [16,17,18,19], and other field crops such as soybean and cotton [20,21], and tomato [22,23] and other vegetable crops such as broccoli, cucumber, melon, and sweet pepper [24,25,26,27]. Canopy reflectance diagnostic tools are classified as passive (e.g., MSR5/87/16R, CropScan Inc., Rochester, MN, USA) or active (e.g., GreenSeeker, Trimble Inc., Sunnyvale, CA, USA) sensors depending on whether the sensor is fitted or not with an own light source, respectively. For passive reflectance sensors, uniform irradiance conditions are highly recommended [28]. 

As a general rule, the adoption of multispectral radiometry is based on the application of Vegetation Indices (VIs) that translate instrument readings into indicators of the actual N or chlorophyll content of the crop. Cui et al. [29] reported the high correlation existing between NDVI [Normalized Difference Vegetation Index: NDVI = (NIR – Red) / (NIR + Red)] and leaf chlorophyll content on tomato plants, thus defining the index as a useful tool to detect nitrogen stress on this crop. Another work on processing tomato [22] showed the relationship between key VIs and main crop features (chlorophyll and N content, N supply and predicted yield), and proposed both NIR/560 [ratio between reflectance readings: NIR/560 = R810 nm wavelength (NIR) / R560 nm wavelength (Green)] and G-NDVI [Normalized Difference Vegetation Index on Greenness: G-NDVI = (NIR – Green) / (NIR + Green)] as the most reliable indicators for the N status of this crop. Padilla et al. [23], in a research carried out on indeterminate tomato grown in a plastic greenhouse, confirmed NDVI as a sensitive indicator of crop N status throughout the crop cycle, being strongly and consistently related to the crop Nitrogen Nutrition Index (NNI), which is the ratio between actual crop N content and the critical crop N content (i.e., the minimum N content necessary to achieve maximum growth). Padilla et al. [23] found that other VIs (G-NDVI, NIR/Green and NIR/Red) were related to crop NNI, but there was less consistency in R^2^ between phenological phases. The strong relationship between VIs and NNI allows obtaining threshold values of these indices, thus facilitating on-farm use of optical sensors to monitor crop N status and optimizing N management.

Precise and quick measurements are the main conditions for both reducing incidence of measurement errors and enabling a timely fertilizer supply. However, each plant monitoring method is characterized by a certain degree of inaccuracy, caused by instrumental bias, operator approach, sampling procedure, as well as environmental and agronomical factors. Several studies have been carried out to understand the variability induced by environmental factors on VIs, which were applied to address vegetation biomass [30]. Nevertheless, little research has been focused on influence of other factors such as agronomical ones (e.g., soil water content, use of organic or plastic mulching, weed presence) on VI reliability. Since optical tools measure gross crop–soil ability to reflect incident light, the presence of reflecting material on background –— such as a plastic mulching film –— may interfere with instrumental readings. It is well known that mulching materials influence soil temperature, wind speed at soil surface, soil water evaporation, soil N availability, as well as crop yield, and literature has shown that plastic-mulched soil can be detected by remote sensing [31,32,33,34,35]. However, no reference is to date available on possible disturbances of soil mulching on the canopy reflectance assessment of crops. 

Furthermore, besides variations in background color, changes in crop pigmentation throughout season, e.g., for presence of mature red fruits, can also affect the reliability of the VIs adopted. Plant redness and its relationship with greenness and NIR have been successfully adopted for assessing the plant nutritional status of tomatoes [22], although the influence of red fruits on radiometric readings has not yet been adequately considered. However, if VIs may be significantly affected by fruit presence, the applications of optical sensors should take into account this source of variation. Since canopy color (chlorophyll and other pigments, including fruit carotenoids) is cultivar-dependent [12], different genotypes should also be considered in assessing VI consistency.

Use of radiometers at different times of the day can generate different VIs in so far as sun angles affect the quality and quantity of light reflected from crop canopies. Few studies have been conducted to determine the effects of sun angles (hour of the day) on the canopy reflectance of crops [36,37,38,39]. Lord et al. [36] studied the effect of sun angles on reflectance in the red and near-infrared regions on canopies of wheat, barley, corn, and sunflower. They found that changes in sun angles had a greater effect on reflectance in the red region than in the NIR region. De Souza et al. [37] demonstrated that spectral radiometer reflectance measurements in corn were influenced by the time of day, and to a lesser extent by sun angles (morning and afternoon measurements were often different at the same sun angle). Ishihara et al [38] found that in general the vegetation indices of rice, corn, and grass decreased with decreasing solar zenith angles (i.e., close to solar noon). Guan and Nutter [39] showed that the percentage of sunlight reflected from alfalfa canopies in all spectral regions obtained prior to 11:00 h and after 15:00 h were significantly higher than reflectance measurements obtained between 11:00 h and 15:00 h. 

Another factor that can affect the canopy reflectance of crops is sensor height. However, very few quantitative studies have been conducted using hand-held multispectral radiometers and, generally, the considered height ranged from 1.5 to 4 m [39]. In a study on cotton canopy reflectance [21] a CropScan MSR 16 spectroradiometer was either positioned either 0.50 or 0.80 m above the canopy, but the two positions were not compared.

This work investigates the stability of several vegetation indices –— the key VIs considered in a previous study [22] –— in assessing canopy reflectance of a processing tomato crop, against some of the above-mentioned factors described as sources of variation. Accordingly, besides increasing N supply, the elements analyzed in this paper are (a) what is the optimal height of the instrument above the canopy (i.e., size of sampling area) to provide useful information on crop canopy reflectance, (b) what sample size is required (i.e., how many individual measures are needed) to successfully monitor a tomato field and what multispectral reading variations are expected, (c) when to sample (i.e., hour of the day) in relation to irradiance conditions during daytime, (d) how consistent the measurements are between different cultivars or in the presence/absence of mulching film or in the presence/absence of maturing fruits.

## 2. Materials and Methods

### 2.1. Experimental Design

Five experiments were carried out in two locations of northern Italy, namely Legnaro (PD), 45°20′ N; 11°57′ E; 8 m a.s.l. and Codigoro (FE), 44°49′ N, and 12°06′ E, 2 m a.s.l., between 2002 and 2005. In all experiments, soil preparation was provided by ploughing (0.35 m depth) plus several harrowings (disk + flexible + spike harrowing). Pest and weed controls were conducted using practices normally adopted by the farmers of the area. The specific features of each experiment were as follows.

*Experiment 1#: Influence of nitrogen supply and height of the multispectral radiometer on main vegetation indices.* The trial was conducted in PD on a silty-loam soil fertilized with a basal dressing of 150 kg phosphorous (P) ha^−1^ and 150 kg potassium (K) ha^−1^. Plants (cv Perfect Peel, Petoseed, Saticoy, CA, USA) were transplanted in single rows on 11 May 11 2002, with a planting density of 33,000 plants ha^−1^ (1.0 m between rows, and 0.3 m spacing between plants). Four N fertilization treatments were considered, 0, 100, 200, and 300 kg N ha^−1^, applied 50% before transplanting and 50% top dressed at the 5th–7th leaf stage (N as ammonium nitrate, NO_3_NH_4_, 34% N). At 65 days after transplanting (DAT), multispectral readings were collected by placing the instruments either at 0.50 or 1.80 m above the canopy, as previously adopted for cotton, wheat, and alfalfa by Bronson et al. [21], Vouillot et al. [40] and Guan and Nutter [39], respectively. The experimental design was a randomized block design with two replicates with the individual plot being 5 × 4 m^2^ (20 m^2^).

*Experiment 2#: Influence of plastic mulching on main vegetation indices throughout the growing season.* In order to assess whether and how soil mulching material would affect overall crop reflectance, a trial was conducted in PD on a silty-loam soil fertilized with a basal dressing of 150 kg phosphorous (P) ha^−1^ and 150 kg potassium (K) ha^−1^. Plants (cv Perfect Peel) were transplanted in paired rows on 24 April 2002, with a planting density of 50,000 plants ha^−1^ (1.2 m between adjacent pairs of rows, 0.4 m between paired rows, and 0.25 m spacing between plants). Nitrogen, as ammonium nitrate (NO_3_NH_4_, 34% N), was applied before transplanting at the rate of 180 kg N ha^−1^. The influence of mulching on the performances of selected VIs was assessed through a comparison between a crop grown over a black PE plastic film and a bare soil control. Radiometer measures were performed seven times between 41 and 85 DAT. The experimental design was a randomized block design with three replicates with the individual plot being 5 × 4 m^2^ (20 m^2^).

*Experiment 3#: Influence of nitrogen supply and hour of sampling on main vegetation indices.* The trial was conducted in PD on a silty-loam soil fertilized with a basal dressing of 150 kg phosphorous (P) ha^−1^ and 150 kg potassium (K) ha^−1^. Plants (cv Perfect Peel) were transplanted in single rows on 23 April 2003, with a planting density of 30,000 plants ha^−1^ (1.0 m between rows, and 0.33 m spacing between plants). Six N fertilizations were studied, 0, 32.5, 65, 130, 195, and 260 kg N ha^−1^, applied 50% before transplanting and 50% top dressed at 5th–7th leaf stage (N as ammonium nitrate, NO_3_NH_4_, 34% N). At 68 DAT, 18 radiometer measurements were performed at different hours of the day (respectively 8:00; 11:00; 14:00; 17:00), in order to verify if the radiometer measurements were stable across the day. The experimental design was a randomized block design with three replicates, with the individual plot being 5 × 4 m^2^ (20 m^2^).

*Experiment 4#: Influence of presence of red fruits on main vegetation indices.* In order to assess whether and how the presence of red fruits would affect overall crop reflectance, a trial was conducted in PD on a silty-loam soil fertilized with a basal dressing of 150 kg phosphorous (P) ha^−1^ and 150 kg potassium (K) ha^−1^. Plants (cv Perfect Peel) were transplanted in single rows on 11 May 2004, with a planting density of 30,000 plants ha^−1^ (1.0 m between rows, and 0.33 m spacing between plants). Nitrogen, as ammonium nitrate (NO_3_NH_4_, 34% N) at the dose of 180 kg N ha^−1^, was applied 50% before planting and 50% top dressed at 5th–7th leaf stage. The influence of the presence of red fruits was assessed by conducting radiometric measures on the same plants just before and after the removal of red fruits, by avoiding disturbance of plant architecture through gentle fruit picking. Measures were taken at 107 DAT. The experimental design was a randomized block design with four replicates with the individual plot being 5 × 4 m^2^ (20 m^2^).

*Experiment 5#: Influence of different cultivars and sample size on main vegetation indices.* The trial was conducted in FE, in a commercial field of 5.4 ha, on a clay soil fertilized with a basal dressing of 130 kg of 10-34-0 N-P-K fertilizer. Eight cultivars were grown, each one extending to 0.6–0.7 ha. The cultivars were: (1) Davis UC82, Agro Parma, Parma, Italy; (2) Early Magnum and (3) UG3002, Unigen seeds, Hollister, CA, USA; (4) ES22-03, EsaSem, Verona, Italy; (5) Falco rosso, Nunhems, Nunhem, Holland; (6) DRI5406, (7) DRI7485, and (8) 83G38, De Ruiter, Holland. Direct sowing was arranged in paired rows on 5 April 2005, to obtain a planting density of 50,000 plants ha^−1^ (1.2 m between adjacent pairs of rows, 0.4 m between paired rows, and 0.25 m spacing between plants). Nitrogen, as urea (46% N) at the dose of 46 kg N ha^−1^, was applied top dressed at the 3rd–4th leaf stage. After approximately 3 weeks, a fertigation program started providing small supplies of N-P-K and Ca every 1 to 3 days up to the beginning of fruit ripening. The fertilizers used (around 10 kg ha^−1^ of fertilizer, each supply) were calcium nitrate (16% N), 14-34-12 N-P-K, 14-10-14 N-P-K, 9-15-38 N-P-K, and 18-18-18 N-P-K. The different types of fertilizer were chosen according to phenological stage and provided a total amount (basal dressing + top dressing + fertigation) of 120 kg N ha^−1^, 100 kg P ha^−1^, 100 kg K ha^−1^, and 45 kg Ca ha^−1^.

On fourteen dates (every five days, from 58 to 123 days after sowing, DAS), ten radiometer readings for each cultivar were performed during a clear sunny day. Influence of sample size on variance reduction was evaluated using bootstrap analysis, as reported below. In addition, 44 measurements on the same plot (cv Davis UC82) were taken at 68 DAS in order to verify the coefficient of variation obtained by the bootstrap procedure (see details below). 

### 2.2. Reflectance Readings and Vegetation Indices Adopted

Reflectance measurements were collected using a handheld Multispectral Radiometer MSR5/87/16R (CropScan Inc., Rochester, MN, USA). The instrument utilizes narrowband interference filters to select discrete bands in the visible and NIR regions of the electromagnetic spectrum. Eight bands were measured within the 460 to 810 nm range, and the following VIs—the same considered in a previous study [22]—were calculated: seven simple wavelength reflectance ratios (NIR/R460, NIR/R510, NIR/R560, NIR/R610, NIR/R660, NIR/R710, NIR/R760), seven normalized indices (Normalized Difference Vegetation Index, NDVI; Normalized Difference Vegetation Index on greenness, G-NDVI; Modified Chlorophyll Absorption in Reflectance Index, MCARI; Soil Adjusted Vegetation Index, SAVI; Optimized Soil Adjusted Vegetation Index, OSAVI; Transformed Soil-Adjusted Vegetation Index, TSAVI; Transformed Chlorophill Absorption in Reflectance Index, TCARI), and two combined indices (TCARI/OSAVI; MCARI/OSAVI). A complete description of the indices as well as of the instrument functioning principles is included in Gianquinto et al. [22]. All plot data were collected as close to solar noon as possible (excluding the experiment on sampling time), on sunny days, clear sky, with absence of clouds or shadow. Plants were in a healthy nutritional status at the time measurements were taken, showing a green color in their leaves.

### 2.3. Data Analysis

The definition of the sample size was achieved by assessing the variability of a complete set of data along several sampling days and on different varieties (Experiment 5#) through the bootstrap procedure [41,42]. Collected data for each VIs were then normalized for varieties and for sampling days. Three data sets were reconstructed.

SET DAY—For each variety, within each day, the average value was calculated (Equation (1)), and then the values obtained were divided by each replicate within the day and the corresponding variety (Equation (2)), and then multiplied by the average of the day (Equation (3)). In this way we obtained a set of data for each day in which the differences were only due to the difference in the replicates (independent from the variety). 

SET VARIETY—For each day, within each variety, the average value was calculated (Equation (4)), and then the values obtained were divided by each replicate within the variety and the corresponding day (Equation (2)), and then multiplied by the variety (Equation (5)). In this way we obtained a set of data for each variety in which the differences were only due to the difference in the replicates (independent from the day).

SET WHOLE DATA—The values obtained with the “SET DAY” procedure (Equation (3)) were divided by the average of each single day (Equation (1)), and multiplied by the average of the whole set of data (Equation (6)). In this way we obtained a set of data (Equation (7)) in which the only source of variability were the differences in the replicates regardless of variety and of day.

(1) $$\bar{X}d_{i}=\frac{\sum_{j=1}^{m}\sum_{k=1}^{p}x_{i,j,k}}{k*j}$$

(2) $$\bar{X}vd_{i,j}=\frac{\sum_{k=1}^{p}x_{i,j,k}}{k}$$

(3) $$Xd_{i,j,k}=x_{i,j,k}*\frac{\bar{X}d_{i}}{\bar{X}vd_{i,j}}=x_{i,j,k}*\frac{\frac{\sum_{j=1}^{m}\sum_{k=1}^{p}x_{i,j,k}}{k*j}}{\frac{\sum_{k=1}^{p}x_{i,j,k}}{k}}=x_{i,j,k}*\frac{\sum_{j=1}^{m}\sum_{k=1}^{p}x_{i,j,k}}{j*\sum_{k=1}^{p}x_{i,j,k}}$$

(4) $$\bar{X}v_{i}=\frac{\sum_{i=1}^{n}\sum_{k=1}^{p}x_{i,j,k}}{k*i}$$

(5) $$Xv_{i,j,k}=x_{i,j,k}*\frac{\sum_{i=1}^{n}\sum_{k=1}^{p}x_{i,j,k}}{i*\sum_{k=1}^{p}x_{i,j,k}}$$

(6) $$\bar{X}w=\frac{\sum_{k=1}^{p}\sum_{i=1}^{n}\sum_{j=1}^{m}x_{i,j,k}}{k*i*j}$$

(7) $$Xr_{i,j,k}=\frac{Equation\;3}{Equation\;1}*Equation\;6$$

with:

*i* = index for the sampling day;
*j* = index for the varieties;
*k* = index for the replicates;
*n* = number of days;
*m* = number of varieties;
*p* = number of replicates;
$X_{i,j,k}$ = value of the considered variable (VI index) in the sampling day i^th^, for the variety j^th^ and for the replicate k^th^;
$\bar{X}d_{i}$ = mean value of the day i^th^;
$\bar{X}v_{j}$ = mean value of the variety j^th^;
$\bar{X}w$ = mean value of the whole experiment;
$\bar{X}vd_{i,j}$ = mean value of the variety j^th^ in the day i^th^;
$Xd_{i,j,k}$ = normalized value, over the effect of varieties, of the considered variable (VI index) in the sampling day i^th^, for the variety j^th^ and for the replicate k^th^;
$Xv_{i,j,k}$ = normalized value, over the effect of sampling day, of the considered variable (VI index) in the sampling day i^th^, for the variety j^th^ and for the replicate k^th^;
$Xr_{i,j,k}$ = normalized value, over the effect of sampling day and variety, of the considered variable (VI index) in the sampling day i^th^, for the variety j^th^ and for the replicate k^th^.

The bootstrap with replacement procedure [41,42] was applied on the data coming from the normalizations described above. Ten thousand subsets of n (from 2 to 100) samples were randomly generated and the corresponding coefficients of variation (CV%) of the samples were calculated. This procedure was applied for each VI separately. Even if the CV% of the samples are not unbiased estimators for small samples [43], this parameter was used to obtain a unique index for comparing small and large samples generated with the bootstrap procedure. The CV% generated were sorted and the 0.025 and 0.975 percentile limits were calculated. In this way it was possible to calculate the 95% confidence interval for the population CV% [41,42,44].

For all other determinations, data were analyzed with Multifactor ANOVA with repeated measures using Statgraphics 15 (Statpoint Technologies Inc., Warrenton, VA, USA) and means were compared using Tukey’s honestly significant difference (HSD) procedure (*p* < 0.05%). The variables DAT (days after transplanting) and DAS (days after sowing) were used as a factor in the multifactor analysis of variance, thus, allowing the evaluation of the interaction between DAT and mulching in Experiment 2# and DAS and tomato varieties in Experiment 5#. 

In Experiment 3#, an analysis of linear regression was performed between N fertilization rates and the VIs responses using Microsoft Excel^®^ Professional Plus 2010. The significance of the linear regression model was tested using Multifactor ANOVA (Statgraphics 15-Statpoint Technologies Inc., Warrenton, VA, USA).

## 3. Results

### 3.1. Experiment 1#: Influence of Nitrogen Supply and Height of the Multispectral Radiometer on Main Vegetation Indices

The instrument position over the crop canopy determines the area measured, which is a circle with a diameter equal to one half of the instrument height. In Experiment 1#, two instrument positions (0.50 and 1.80 m in height, corresponding to a sampling area of about 0.05 and 0.64 m^2^, respectively) were compared, versus four N supplies (0, 100, 200, and 300 kg ha^−1^). Measures were conducted at 65 DAT, during the “full fruit growth” phenological stage (Table 1), which generally corresponds to the period of maximum peak for N uptake of the tomato crop. 

At sampling, both nitrogen rate and instrument height significantly affected the reflectance for all the considered wavelengths (Table 2). 

**Figure 1:**
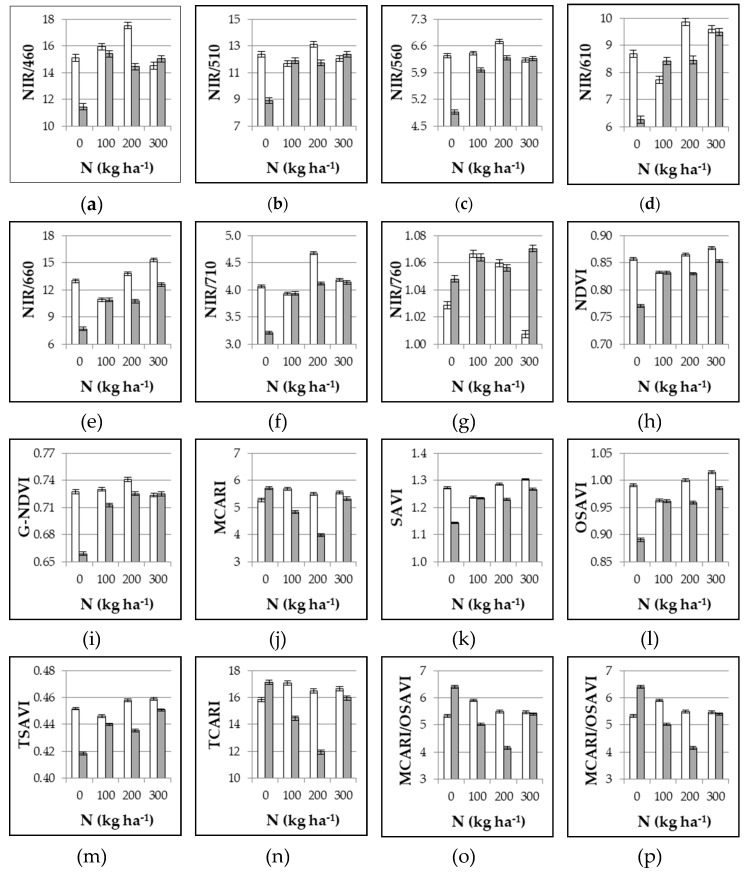
Experiment 1#. Influence of nitrogen supply (0, 100, 200, 300 kg ha^−1^) and position of multispectral radiometer (0.5 m height white bars, and 1.8 m height grey bars) on different VIs as assessed at 65 DAT. Mean values ± SE.

The unfertilized control showed the highest reflectance for all the bands within the visible range and up to 710 nm, with increases of 14.5%, 12.6%, 10.6%, 17.3%, 21.5%, and 11.7% as compared with the mean of the N fertilized plots at 460, 510, 560, 610, 660, and 710 nm, respectively (data not shown). At near infrared wavelengths, the reflectance of the control was slightly but significantly lower (−1.9% and −3.3% at 760 and 810 nm, respectively, data not shown). By positioning the instrument closer to the crop, the reflectance was higher all along the light spectrum, with the exclusion of 660 nm where reflectance was lower. As compared with 1.80 m height, the percent variation was +3.4%, +5.4%, +5.4%, +5.4%, –8.4%, +6.3%, +19.5% and +17.4% at 460, 510, 560, 610, 660, 710, 760, and 810 nm, respectively (data not shown). As a consequence, all the derived VIs were significantly affected by both N and height treatments, and significant interaction effects were always observed (Table 2). When instrument was positioned at 1.80 m high, VIs increased in N fertilized treatments as compared with unfertilized control, except for MCARI, TCARI, and the combined indices (TCARI/OSAVI and MCARI/OSAVI) where the highest values were shown in absence of N fertilization (Figure 1). The VIs response to intermediate N doses (100 and 200 kg ha^−1^, in this experiment) is of main relevance, since it is within these values (rather than the extreme ones, unlikely to occur in the field) that the practical N application occurs. Accordingly, the best indices were NIR/R560, NIR/710, and G-NDVI, as they were the only VIs able to discriminate among these levels of N fertilization, with significant increases when fertilization was augmented from 100 to 200 kg ha^−1^ (Figure 1). Moving the instrument closer (0.5 m height), the VIs appeared to be frequently erratic without consistent relationships with N rates. 

### 3.2. Experiment 2#: Influence of Plastic Mulching on Main Vegetation Indices Throughout the Growing Season

In Experiment 2#, plastic mulching influence on instrument performance was assessed. Consistently, reflectance at all measured wavelengths (Table 3) was diminished by black PE film, and the effect on each band varied across the growing season being largely more evident up to 71 DAT (data not shown). Considering the mean values between 41 and 71 DAT, the reflectance of the mulched crop was reduced by 37%, 39%, 46%, 52%, 54%, 56%, 48%, and 50% at 460, 510, 560, 610, 660, 710, 760, and 810 nm, respectively. In the remaining 2 weeks, the wavelengths that were affected more by mulching were 610, 660, 710 nm (33–35% reduction), followed by 510 and 560 nm (23–27%), while 460, 760, and 810 were the least penalized by the PE film (16–18% reduction) (data not shown).

**Figure 2:**
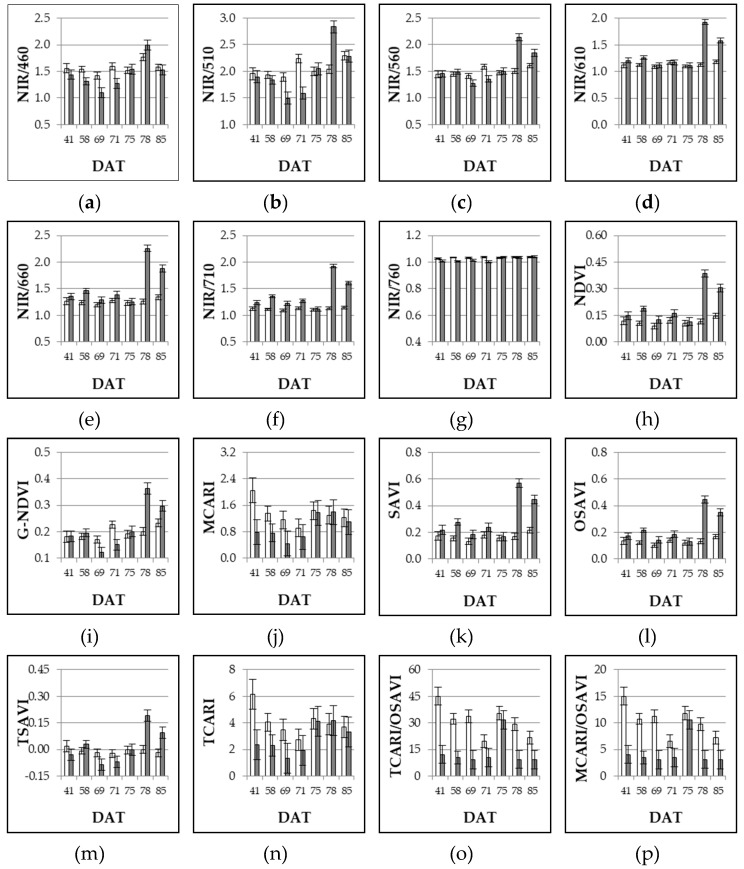
Experiment 2#. Influence of bare soil (white bars) and black mulching film (grey bars) on different VIs as assessed throughout the growing season (41, 58, 69, 71, 75, 78, 85 DAT). DAT = days after transplanting. Mean values ± SE.

These effects on light bands translated into the derived VIs (Table 3, Figure 2), most of them showing significant differences between mulched and not mulched crop on relatively few sampling dates (three-four out of seven), while only NIR/710, TCARI/OSAVI and MCARI/OSAVI were affected almost all along the season (six observations out of seven). Moreover, for most of the VIs, relevant differences, in terms of magnitude, occurred during the last part of the season, while NIR/460, MCARI, TCARI, and combined TCARI/OSAVI and MCARI/OSAVI showed the largest differences during the first half of the sampling period.

### 3.3. Experiment 3#: Influence of Nitrogen Supply and Hour of Sampling on Main Vegetation Indices

During Experiment 3#, VIs were assessed against daily time of sampling using four sampling times (8:00, 11:00, 14:00, and 17:00), and six N doses (0, 32.5, 65, 130, 195, and 260 kg N ha^−1^). Measures were conducted at 68 DAT, during the “full fruit growth” phenological stage (Table 1). Both nitrogen rate and sampling time significantly affected the reflectance for all the considered wavelengths, while interaction effects “N rate × hour” were never found (Table 4). 

The unfertilized control showed the highest reflectance from 510 up to 710 nm with increases of 11.3%, 8.3%, 14.0%, 15.7%, and 3.8%, as compared with the mean of the N fertilized plots, at 510, 560, 610, 660, and 710 nm, respectively (data not shown). At blue and near infrared wavelengths the reflectance of the control was significantly lower (−14.0%, −14.5%, and −15.8% at 460, 760, and 810 nm, respectively). The reflectance of all the considered wavelengths varied considerably during the day and the highest values were observed either at 8:00 (460, 710, 760, and 810 nm) or at 11:00 (all the other bands), while the lowest ones were always detected at 17:00 with sensible reductions of reflectance, ranging 45–70%, as compared with morning time (data not shown).

**Figure 3:**
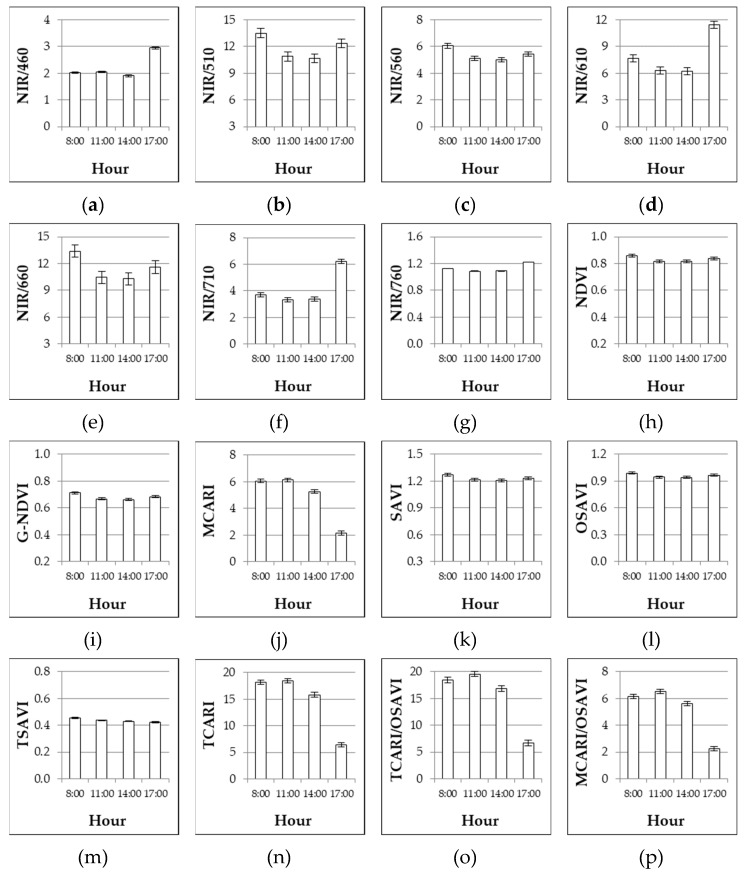
Experiment 3#. Influence of the hours of the day on different VIs as assessed at 68 DAT. Mean values ± SE.

In the last column of Table 4, the significance of the regression between nitrogen fertilization rates and the response in term of VIs is included. Most VIs (with the only exception of NIR/460) were significantly correlated with nitrogen fertilization in the processing tomato crop. The highest coefficients of determination (R^2^) were found for NIR/560 and G-NDVI. Observing the VIs’ response to the day’s hour (Table 4, Figure 3), almost all the simple wavelength reflectance ratios and normalized indices showed lower and statistically similar values at 11:00 and 14:00, against higher and different values observed at 8:00 and 17:00 (the magnitude of difference varied index by index). A different response was observed for MCARI, TCARI, and combined indices (TCARI/OSAVI and MCARI/OSAVI) as higher and not different values were observed at 8:00 and 11:00, then they decreased reaching a minimum index at 17:00. 

### 3.4. Experiment 4#: Influence of the Presence of Red Fruits on Main Vegetation Indices

During Experiment 4#, measures were conducted on the same plants just before and after fruit harvest in order to assess key VIs variability induced by ripened fruit. The removal of the fruits significantly affected the reflectance of almost all the wavelengths considered in this study (Table 5). Only the 560 nm band did not show significant differences between treatments. The plots where red fruits were removed showed the highest reflectance, as compared with plants with red fruits, at 460, 510, 610, 660, and 710 nm with increases of 23.6%, 27.7%, 27.1%, 28,3%, and 9.6%, respectively (data not shown). At near infrared wavelengths, the reflectance of the plant without red fruits was significantly lower (−11.6% and −10.8% at 760 and 810 nm, respectively). As a consequence, most of the derived VIs were significantly affected and increased by the presence of red fruits, with the only exception of NIR/560, G-NDVI, TCARI/OSAVI, and MCARI/OSAVI where no differences were noted (Table 5).

### 3.5. Experiment 5#: Influence of Different Cultivars, and Sample Size on Main Vegetation Indices Throughout the Growing Season

In Experiment 5#, both cultivars and date of sampling significantly affected the reflectance for all the considered wavelengths (Table 6). Consistently, all VIs were able to discriminate the main factors—variety (a) and date of sampling (b). Probability was extremely high for both Factor (a) and Factor (b)—P tended to zero and was non-computable with standard spreadsheets or statistical packages—and for the interaction (a) × (b).

The determination of the sample size useful to provide further significant information in an experiment is crucial for the correct application of diagnostic non-destructive tools. In Experiment 5# we used the bootstrapping with replacement procedure in order to estimate the coefficients of variation of population (CV%) of the measurements of VIs and identify the most suitable number of observations to have a reliable assessment.

**Figure 4:**
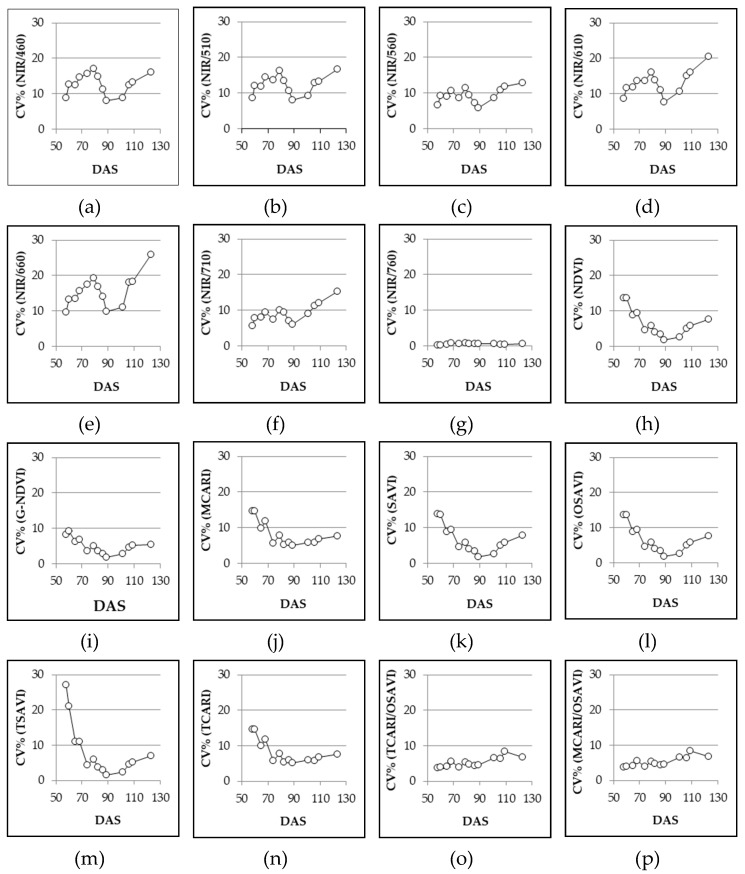
Experiment 5#. Coefficient of variation (CV%) of different VIs across different sampling dates, generated with bootstrap analysis by using SET DAY (see Section 2.3 data analysis). DAS = days after sowing.

When we look at Figure 4, it is possible to observe the effect of sampling dates on the VIs (using SET DAY data). Among the VIs considered in this study, the simple wavelength reflectance ratios (from NIR/460 to NIR/760) were shown to be more unstable, as the range of the CV% found using the bootstrap procedure had higher results than in both the normalized (from NDVI to TCARI) and the combined VI_S_ (TCARI/OSAVI and MCARI/OSAVI) indices. The only exception was NIR/760 and, partially, NIR/710. Among simple wavelength reflectance ratios, the highest variability was observed for NIR/660.

**Figure 5:**
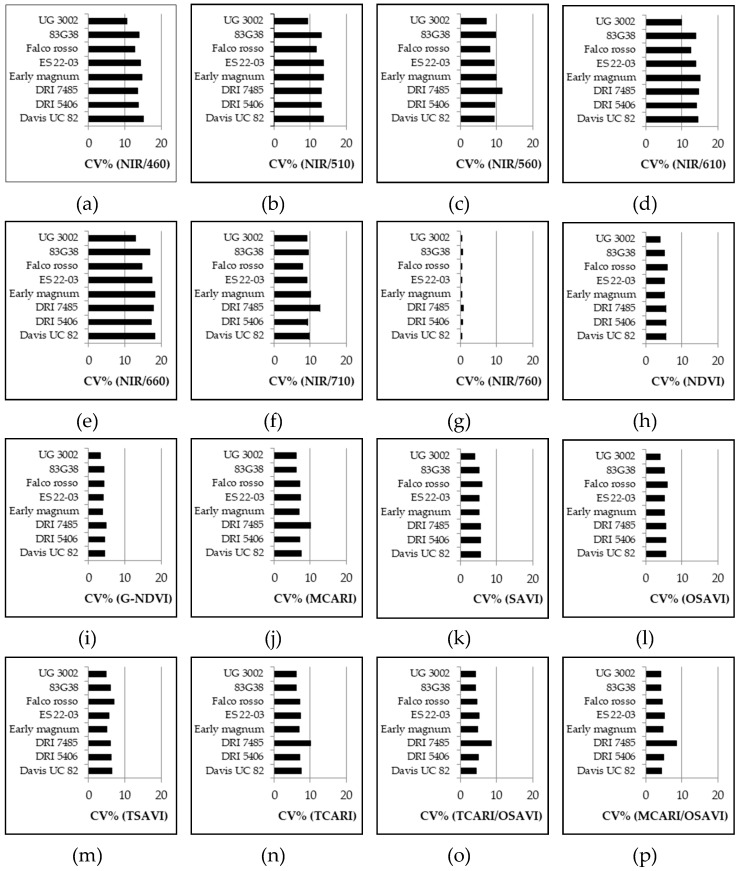
Experiment 5#. Coefficient of variation (CV%) of different VIs for different varieties, generated with bootstrap analysis by using SET VARIETY (see Section 2.3 data analysis).

Considering the VARIETY SET data (Figure 5), the CV% showed a great stability within varieties even if the data came from all measurements throughout the growing season. Among the other simple wavelength reflectance ratios, the NIR/560 result was the least influenced by variety and the CV% was relatively low, while the VI with the highest CV% was again NIR/660. For the normalized and combined VIs all the values were relatively low but G-NDVI was one of the most stable VIs among varieties, while MCARI and TCARI presented the highest CV%. Only one variety, DRI 7485, presented higher CV% for many VIs (almost half of the considered VIs), but the variations were not so high. 

**Figure 6:**
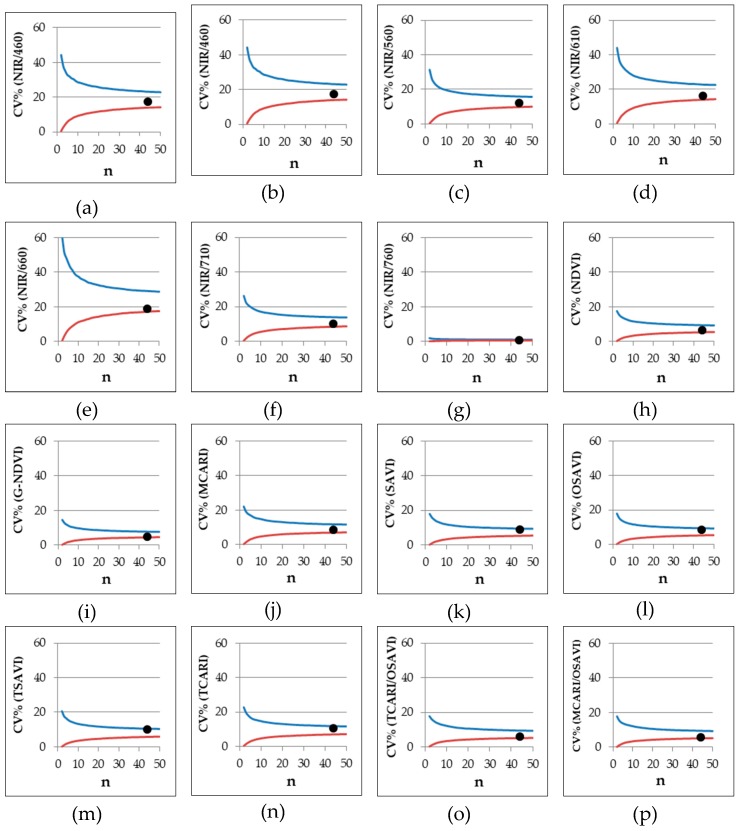
Experiment 5#. Coefficient of variation (CV%) limits (P = 95%) of different VIs with number of observations ranging 2–50 observations per measurement, generated with bootstrap analysis by using SET WHOLE data (see Section 2.3 data analysis) (red line, lower limit at P = 2.5%; blue line, upper limit at P = 97.5%). The filled circles represent the CV% of a set of 44 data taken for the variety Davis UC82 as assessed at 68 DAS.

From the SET WHOLE data (Table 7, Figure 6) is possible to observe the variability of the replicates regardless of the variety and of the date of sampling, and consequently the change of CV% related to the size of the data set. In general, for NIR/760, normalized, and combined VIs, a set of 5 data (n = 5) is sufficient to have reliable output with a CV% always below 20%. Among normalized VIs, G-NDVI proved to be the best, already showing an upper limit CV% (0.975 percentile limit) lower than 10% with n = 10. Good performances were also shown by NDVI, SAVI, and OSAVI. Considering the other simple wavelength reflectance ratios, good results were also given by NIR/710 and NIR/560, both displaying upper limit CV% less than 20% with n = 10. For the other VIs, upper limit CV% never fell below 20% and the CV% range was wider.

The bootstrap procedure, as performed in our experiment, can be seen as a tool for generating an enormous amount of data; in other words, an artificial set of data with low correspondence to the initial data set. In order to verify and exclude this eventuality—or refuse this hypothesis—a set of 44 data was taken for the variety Davis UC82 at 68 DAS—around the start of flowering stage—and the CV% for each VI was calculated. The values calculated for Davis UC82 were always within the 95% variation limits (Figure 6, filled circles) proving the reliability of the adopted procedure.

## 4. Discussion

The correct use of multispectral radiometers can give a quick and accurate estimation of the N status of tomato crops [22,23]. Our previous work [22] demonstrated that the simple wavelength reflectance ratio NIR/560 is a strong predictive index for crop N status, as it appeared to be the best indicator of leaf chlorophyll concentration, as well as of leaf N concentration (together with NIR/610). Moreover, it proved to be able to discriminate among N fertilization (together with NIR/510, NIR/710, and G-NDVI), allowing the detection of even little variations in N supply (together with G-NDVI). Finally, together with G-NDVI, it appeared to be the best indicator for yield prediction, being able to also discriminate between small yield variations [22]. 

In this work, the consistency of both NIR/560 and G-NDVI in discriminating among N fertilization has also been confirmed by the dramatically higher R^2^ values found for the linear regression line at increasing N rates tested in Experiment 3#. On the other hand, the results obtained in Experiment 1# confirmed that NIR/560, NIR/710, and G-NDVI are the most accurate VIs, able to reveal variations in intermediate N doses, this being actually relevant as it is within the intermediate rates (rather than extreme ones, unlikely to occur in field) that practical N application occurs.

It was also previously suggested that many other environmental and agronomic factors affect a crop’s total canopy reflectance [2], including, e.g., light intensity, presence of diseases and nutrient disorders not related to a crop’s N status, soil water content, use of organic or plastic mulching, weed presence, different varieties. This, together with a certain degree of inaccuracy—caused by instrumental bias, operator approach, sampling procedure—inevitably lead to an increase in measurements error. Accordingly, this work addressed issues related to standardization of measuring procedures, as well as stability of VIs in presence of some “disturbing elements”.

In order to achieve reliable readings, it is possible to estimate, at field scale, data variability obtainable by using the bootstrap procedure and this can be used for further determination of the sample size required in an experiment [44]. The relationship between sample size and the ability of the Cropscan MSR5 field spectroradiometer to assess both diversity and productivity of a grassland was stressed in a study by Csillag et al. [45]. Although their study was carried out over a natural ecosystem rather than an agricultural field, they stated that a reliable correlation between sensor readings and both species composition and yield would be found when basing the sample size on the maximum number of species combinations. On the other hand, the application of multispectral radiometry over agricultural fields has been successfully applied by Vouillot et al. [40] on wheat, with significant correlation from using two independent readings per plot. Afterward, experiments on corn [17] considered two measures per plot sufficient to provide reliable indications. Furthermore, as far as sample size is concerned, in some works on potato, an acceptable coefficient of variation was provided by 3 randomized measurements in each experimental plot [46,47]. In this paper, we demonstrated that measurements of all normalized and combined VIs had a very low range of variability in the coefficients of variation—since they are normalized indices—and a minimal number of replicates (n = 5) is recommended when a coefficient of variation lower than 20% is required. Among normalized VIs, G-NDVI showed the best performance with a coefficient of variation lower than 10% when a number of replicates equal to 10 was provided. Furthermore, a limited variability in NIR/760 (CV < 20% when n = 5) was also observed. This may be ascribed to the proximity between the spectral regions considered in the ratio (e.g., 760 and 810), which resulted in index values proximal to 1. A number of replicates around 10 appeared to be the minimum to have an acceptable coefficient of variation (lower than 20%) for NIR/710 and NIR/560. All the other simple wavelength reflectance ratios appeared less reliable, showing a greater variability. Among these, the highest variability was observed for NIR/660 (Figure 6), which was therefore characterized by a high degree of uncertainty. Nevertheless, the light absorbance/reflectance in the red zone is strictly linked to quantum yield for CO_2_ uptake [48]. Consistently, NIR/660 could be used as good discriminating index of photosynthetic efficiency, but more specific research should be addressed on this topic.

Regarding the instrument height, in previous reports a CropScan MSR was either positioned at 0.50 or 0.80 m above the canopy on cotton [21], or between 1.50 and 4.0 m on alfalfa [39], and a CIMEL field radiometer was placed at height of about 2 m over the canopy on wheat [40]. Since the area undergoing multispectral measurement is a circle with diameter that is about half of the height of the instrument, these positions measured either the canopy of an individual plant or part of it (diameter 0.25 or 0.40 m) or the area where few plants are grown (diameter 0.75 to 2 m). This brings us back to the considerations of whether instrument reliability to assess plant N status is more greatly influenced by interferences of soil between the plants or by variability between and/or within plants. Our results have shown how, for a precise and reliable data collection, measures should be performed by keeping the instrument 1.80 m above the soil surface rather than 0.5 m, including more than one plant in order to reduce variability (Figure 1). When the sensor is very close to the plant the detected area is very small and interferences—and high variability—are probably due to the architecture/orientation of branches and leaves that undoubtedly affects reflectance of the light. This disturbance element has shown to be more relevant than soil in the background.

A further experiment (Experiment 3#) was conducted in order to assess the response of VIs to changes in incident radiation along the day, as this issue has been scarcely considered in available literature on passive canopy reflectance sensors. Nevertheless, some works demonstrated that vegetation indices of rice, corn, grass, and alfalfa decreased close to solar noon [38,39]. Other experiences are present in literature for active canopy reflectance sensors revealing that they are able to measure at any time of day regardless of variations in solar irradiance [27,49,50,51]. On the other hand, studies reporting slight effects of the time of day on measurements of active canopy reflectance sensors also exist [28,52,53]. Indeed, as a consequence of different irradiance throughout the day, in our experiment—which used passive canopy reflectance sensors—higher variability was observed at 8:00 and 17:00, as compared to 11:00 and 14:00 when the key VIs were fairly steady (Figure 3). Our results have confirmed the findings on alfalfa [39] other than the manufacturer guidelines [54], thus suggesting the central hours of the day as optimal time to take measurements. Nevertheless, as no significant interaction found with nitrogen doses (i.e., the crop relative response to nitrogen supply did not change during the day), two modus operandi could be hypothesized according to the approach used to interpret sensor measurements [5]: (1) when an absolute approach—based on sufficiency/threshold values—is used, measurements (as well as assessment of the sufficiency/threshold values) should be done at central hours of the day; (2) when a relative approach—based on reference/spy plots—is used, measurements could be done at any time, but at the same time of reference/spy plot.

Black bodies absorb most of the incident radiation [33]. Accordingly, reduced reflectance at all wavelengths in response to black mulching film was observed in Experiment 2#. Nevertheless, the effect translated in a different way into derived VIs and across the growing season (Figure 2). Most of VIs showed significant differences between mulched and not mulched crop in relatively few sampling dates and in the last part of the season, when monitoring is actually less relevant for crop N status assessment and fertilization management. Several VIs (including NIR/460, NIR/710, MCARI, TCARI, TCARI/OSAVI, and MCARI/OSAVI), were significantly affected by mulching during the first part of the growing period, or even along the season.

Besides variations in the background color due to soil mulching materials, changes in crop pigmentation due to the presence of mature red fruits [22] also affected VIs, as shown in Experiment 4# where most of VIs were increased by their presence (Table 5). Nevertheless, the relevance of this finding depends on the N fertilization method adopted for the crop. If crop is supplied with traditional granular fertilizers, the last top dressing generally occurs not later than the full flowering stage, when red fruits are not yet appeared. In this case, there is no disturbance on crop monitoring. If crop is fertigated, N supplies can last till the late fruit growth stage when many red fruits are present. In this case, most VIs are unreliable, excluding NIR/560, G-NDVI, TCARI/OSAVI, and MCARI/OSAVI, which were not influenced by maturing fruits (Table 5).

As stated in the introduction, different genotypes should also be considered in assessing the VIs’ consistency and this was confirmed by the results of Experiment 5#, where significant differences were found among cultivars for all the VIs considered (Table 6). However, the CV% was always below 20% for each of the VIs considered. While this does not exclude that there could be varieties with higher variability, in the present work all indices can be seen as overall stable for each variety. Among simple wavelength reflectance ratios, NIR/560 was less influenced by the variety, with a relatively low CV%, while within normalized and combined VIs, G-NDVI appeared to be one of the most stable among varieties. Comparing these two VIs, the lowest CV% was achieved by the cultivar UG 3002 for both NIR/560 and G-NDVI (Figure 5). In general, the CV% of NIR/560 showed higher ranges than the corresponding CV% of G-NDVI for all the cultivars. This leads to a lower level of accuracy of NIR/560 if a comparison of cultivars is requested. No general trend was observed for cultivars even if lower values of confidence intervals for the CV% of G-NDVI were observed. Both indices had different responses to the day of sampling (Figure 4) but the variability was lower for G-NDVI. Notably, a greater difference between the worst (high variability) and the best (low variability) performance of G-NDVI was observed as compared with corresponding values of NIR/560. The CV% of NIR/560 showed higher ranges than the corresponding G-NDVI along the whole cycle (Figure 6). This leads to reduced precision in the estimation of NIR/560 compared with G-NDVI. For both variables, the minimum range was observed at around 90 DAS (Figure 4). It is interesting to note that for both variables the highest level of variability was observed at the beginning and at the end of the cycle. While the end of season variability is not relevant as no agronomic interventions are performed anymore, in the initial stages of cultivation a more stable measurement could be useful for properly defining, for example, fertilization management [22]. 

## 5. Conclusions

Integrating the results from this work with previous experimental evidences [22], two VIs proved to be the most reliable in predicting nitrogen status and managing nitrogen fertilization of a processing tomato field crop: NIR/560 and G-NDVI, also called the Green Ratio of Vegetation Index, GVI [55], and the Green Normalized Difference Vegetation Index [56], respectively. They were the best indicators of leaf chlorophyll and nitrogen concentration, and showed a great ability in discriminating among nitrogen fertilization rates [57], allowing the detection of even little variations in nitrogen supply. They also appeared to be the best indicators for yield prediction, also giving high precision between small variations in yield. Furthermore, NIR/560 and G-NDVI showed a moderate variability, indicating that a relatively small sample size (5–10 observations) is required to obtain reliable measurements. Measurement consistency is also enhanced when the multispectral radiometer is positioned at such a distance from the crop to allow the detection of several plants, and when measurements are taken during the central hours of the day (if passive canopy reflectance sensors are used). Both NIR/560 and G-NDVI also demonstrated to be unaffected by the appearance of maturing fruits, while most of the others VIs were altered by their red color. Furthermore, changes in NIR/560 and G-NDVI behavior due to the presence of black soil mulching film proved to be almost negligible during the first part of the growing season, when accurate N status assessment is crucial. Ultimately, even though NIR/560 and G-NDVI behavior varied with tomato cultivar, they appeared the most stable among VIs. Since VI behavior is cultivar dependent, an added value for seed companies should be characterizing and providing specific VI patterns for each cultivar, thus allowing producers to adopt precision farming fruitfully.

## Figures and Tables

**Table 1:** Phenological stages for a processing tomato crop grown in Northern Italy (Gianquinto, personal communication).

Planting Method		Phenological Stage
Transplanting	Direct Sowing	
Weeks After Transplanting ^1^	Weeks After Sowing ^1^	
–	1–3	Emergence
3–4	4–7	30% soil cover
4–5	7–10	7th true leaf (start of fast growth)
5–6	9–11	Start of flowering
7–8	11–15	Start of fruit growth
9–10	13–17	Full fruit growth
11–12	15–19	Start of ripening
12–13	16–20	30% fruit ripening
14–15	18–25	Fruit harvest

^1^ In relation to season and cultivar.

**Table 2:** Experiment 1#. Statistical significance of each of the factors (N rate, and instrument height) and of the interaction between factors by ANOVA. * *p* < 0.05, ** *p* < 0.01, *** *p* < 0.001, NS not significant.

	N Rate (a)	Instrument Height (b)	(a) × (b)
**Wavelength**			
460	***	***	***
510	***	***	***
560	***	***	***
610	***	***	***
660	***	***	***
710	***	***	***
760	***	***	***
810	***	***	***
**Simple Wavelength Reflectance Ratios**			
NIR/460	***	***	***
NIR/510	***	***	***
NIR/560	***	***	***
NIR/610	***	***	***
NIR/660	***	***	***
NIR/710	***	***	***
NIR/760	***	***	***
**Normalized Indices**			
NDVI	***	***	***
G-NDVI	***	***	***
MCARI	***	***	***
SAVI	***	***	***
OSAVI	***	***	***
TSAVI	***	***	***
TCARI	***	***	***
**Combined Indices**			
TCARI/OSAVI	***	***	***
MCARI/OSAVI	***	***	***

**Table 3:** Experiment 2#. Statistical significance of each of the factors (plastic mulching, and date of sampling) and of the interaction between factors by ANOVA. * *p* < 0.05, ** *p* < 0.01, *** *p* < 0.001, NS not significant.

	Plastic Mulching (a)	Date of Sampling (b)	(a) × (b)
**Wavelength**			
460	***	***	***
510	***	***	***
560	***	***	***
610	***	***	***
660	***	***	***
710	***	***	***
760	***	***	***
810	***	***	***
**Simple Wavelength Reflectance Ratios**			
NIR/460	***	***	***
NIR/510	***	***	***
NIR/560	***	***	***
NIR/610	***	***	***
NIR/660	***	***	***
NIR/710	***	***	***
NIR/760	***	***	***
**Normalized Indices**			
NDVI	***	***	***
G-NDVI	***	***	***
MCARI	***	***	**
SAVI	***	***	***
OSAVI	***	***	***
TSAVI	***	***	***
TCARI	***	***	**
**Combined Indices**			
TCARI/OSAVI	***	***	***
MCARI/OSAVI	***	***	***

**Table 4:** Experiment 3#. Statistical significance of each of the factors (N rate, and hour of the day) and of the interaction between factors by ANOVA, and linear regression analysis between N fertilization rates and VIs responses. * *p* < 0.05, ** *p* < 0.01, *** *p* < 0.001, NS not significant.

	N Rate	Hour of Day	(a) × (b)	VIs = f(N Rate)			
	(a)	(b)		Intercept	Slope	R^2^	Sign.
**Wavelength**							
460	***	***	NS	16.82899	0.01779	0.06340	***
510	***	***	NS	3.60776	−0.00185	0.04334	***
560	***	***	NS	7.73276	−0.00313	0.03131	***
610	***	***	NS	6.06534	−0.00431	0.03822	***
660	***	***	NS	3.89937	−0.00302	0.09675	***
710	**	***	NS	11.10698	−0.00413	0.00928	***
760	***	***	NS	31.58037	0.03351	0.09770	***
810	***	***	NS	35.23383	0.03842	0.11925	***
**Simple Wavelength Reflectance Ratios**							
NIR/460	ns	***	NS	2.21645	0.00008	0.00028	ns
NIR/510	***	***	NS	9.82605	0.01772	0.52784	***
NIR/560	***	***	NS	4.53060	0.00761	0.63670	***
NIR/610	***	***	NS	6.34761	0.01370	0.23411	***
NIR/660	***	***	NS	9.09626	0.02047	0.51980	***
NIR/710	***	***	NS	3.49459	0.00579	0.15447	***
NIR/760	***	***	NS	1.12601	0.00003	0.00272	**
**Normalized Indices**							
NDVI	***	***	NS	0.79966	0.00028	0.48844	***
G-NDVI	***	***	NS	0.63852	0.00038	0.61673	***
MCARI	ns	***	NS	5.07250	−0.00146	0.00647	***
SAVI	***	***	NS	1.18335	0.00043	0.49112	***
OSAVI	***	***	NS	0.92357	0.00033	0.48994	***
TSAVI	***	***	NS	0.41919	0.00014	0.39787	***
TCARI	ns	***	NS	15.21751	−0.00438	0.00647	***
**Combined Indices**							
TCARI/OSAVI	***	***	NS	16.49165	−0.00992	0.02902	***
MCARI/OSAVI	***	***	NS	5.49722	−0.00331	0.02945	***

**Table 5:** Experiment 4#. Statistical significance by ANOVA and mean values of the “red fruit” factor. * *p* < 0.05, ** *p* < 0.01, *** *p* < 0.001, NS not significant.

	Significance	Reflectance (%)	
		Red Fruit	
		No	Yes
**Wavelength**			
460	***	3.30	2.67
510	***	4.38	3.43
560	NS	6.92	7.40
610	***	5.95	4.68
660	***	4.81	3.75
710	***	11.93	10.89
760	***	40.50	45.84
810	***	43.29	48.51
**Simple Wavelength Reflectance Ratios**			
NIR/460	***	13.19	18.15
NIR/510	***	9.95	14.14
NIR/560	NS	6.30	6.56
NIR/610	***	7.34	10.35
NIR/660	***	9.16	12.96
NIR/710	***	3.64	4.45
NIR/760	***	1.07	1.06
**Normalized Indices**			
NDVI	***	0.80	0.86
G-NDVI	NS	0.72	0.73
MCARI	**	4.64	5.10
SAVI	***	1.19	1.27
OSAVI	***	0.92	0.99
TSAVI	***	0.43	0.45
TCARI	**	13.91	15.30
**Combined Indices**			
TCARI/OSAVI	NS	15.05	15.47
MCARI/OSAVI	NS	5.02	5.16

**Table 6:** Experiment 5#. Statistical significance of each of the factors (variety, and date of sampling) and of the interaction by ANOVA. * *p* < 0.05, ** *p* < 0.01, *** *p* < 0.001, NS not significant.

	Variety (a)	Date of Sampling (b)	(a) × (b)
**Wavelength**	***	***	***
460	***	***	***
510	***	***	***
560	***	***	***
610	***	***	***
660	***	***	***
710	***	***	***
760	***	***	***
810	***	***	***
**Simple Wavelength Reflectance Ratios**			
NIR/460	***	***	***
NIR/510	***	***	***
NIR/560	***	***	***
NIR/610	***	***	***
NIR/660	***	***	***
NIR/710	***	***	***
NIR/760	***	***	***
**Normalized Indices**			
NDVI	***	***	***
G-NDVI	***	***	***
MCARI	***	***	***
SAVI	***	***	***
OSAVI	***	***	***
TSAVI	***	***	***
TCARI	***	***	***
**Combined Indices**			
TCARI/OSAVI	***	***	***
MCARI/OSAVI	***	***	***

**Table 7:** Experiment 5#. Coefficient of variation (CV%) limits (P = 95%) with different number of observations per measurement (N = 5, N = 10, and N = 20), generated with bootstrap analysis by using SET WHOLE data (see Section 2.3 data analysis). LL, lower limit at P = 2.5%; UL, upper limit at P = 97.5%.

	N = 5		N = 10		N = 20	
	UL (97.5%)	LL (2.5%)	UL (97.5%)	LL (2.5%)	UL (97.5%)	LL (2.5%)
**Simple Wavelength Reflectance Ratios**						
NIR/460	32.803	5.793	28.616	9.236	25.755	11.716
NIR/510	31.474	5.478	26.777	8.753	23.771	11.235
NIR/560	22.548	3.855	19.243	6.395	17.505	8.376
NIR/610	32.747	5.720	27.990	9.122	25.131	11.936
NIR/660	44.708	6.967	37.206	11.085	32.684	14.463
NIR/710	19.654	3.335	16.875	5.491	15.244	7.031
NIR/760	1.441	0.239	1.270	0.379	1.118	0.487
**Normalized Indices**						
NDVI	13.755	2.044	11.788	3.336	10.475	4.369
G-NDVI	10.956	1.772	9.569	2.831	8.557	3.640
MCARI	16.974	2.893	14.666	4.605	12.980	5.932
SAVI	13.654	2.054	11.704	3.291	10.342	4.339
OSAVI	13.793	2.043	11.760	3.285	10.387	4.334
TSAVI	15.399	2.147	13.206	3.556	11.775	4.759
TCARI	17.019	2.917	14.683	4.604	13.000	5.878
**Combined Indices**						
TCARI/OSAVI	14.024	2.117	11.909	3.306	10.512	4.319
MCARI/OSAVI	13.534	2.168	11.879	3.400	10.544	4.289

## References

[1] Sambo P., Zanin G., Forte V. (2009). Cropscan as a tool to drive phosphorus and potassium fertilization in tomato. Acta Hortic..

[2] Gianquinto G., Orsini F., Sambo P., Paino D’urzo M. (2011). The use of diagnostic optical tools to assess nitrogen status and to guide fertilization of vegetables. HortTechnology.

[3] Katsoulas N., Elvanidi A., Ferentinos K.P., Kacira M., Bartzanas T., Kittas C. (2016). Crop reflectance monitoring as a tool for water stress detection in greenhouses: A review. Biosyst. Eng..

[4] Ihuoma S.O., Madramootoo C.A. (2017). Recent advances in crop water stress detection. Comput. Electron. Agric..

[5] Padilla F.M., Gallardo M., Peña-Fleitas M.T., de Souza R., Thompson R.B. (2018). Proximal optical sensors for nitrogen management of vegetable crops: A review. Sensors.

[6] Ihuoma S.O., Madramootoo C.A. (2019). Sensitivity of spectral vegetation indices for monitoring water stress in tomato plants. Comput. Electron. Agric..

[7] Zhang G., Tao X., Zhang Z., Du Y., Lü X. (2017). Monitoring of Aphis gossypii using Greenseeker and SPAD meter. J. Indian Soc. Remote Sens..

[8] Xia J., Yang Y., Cao H., Ge S., Chen G. (2018). Performance analysis of clustering method based on crop pest spectrum. Eng. Agric. Environ. Food.

[9] Alves T.M., Moon R.D., MacRae I.V., Koch R.L. (2019). Optimizing band selection for spectral detection of *Aphis glycines* Matsumura in soybean. Pest Manag. Sci..

[10] Scharf P.C., Lory J.A. (2002). Calibrating corn color from aerial photographs to predict sidedress nitrogen need. Agron. J..

[11] Gianquinto G., Sambo P., Borsato D. (2006). Determination of SPAD threshold values for the optimisation of nitrogen supply in processing tomato. Acta Hortic..

[12] Gianquinto G., Goffart J.P., Olivier M., Guarda G., Colauzzi M., Dalla Costa L., Delle Vedove G., Vos J., MacKerron D.K.L. (2004). The use of hand-held chlorophyll meters as a tool to assess the nitrogen status and to guide nitrogen fertilization of potato crop. Potato Res..

[13] Gianquinto G., Sambo P., Orsini F., Sciortino M., Forte V. (2006). Optical tools, a suitable means to reduce nitrogen use in fertigated tomato crop. HortScience.

[14] Tremblay N., Fallon E., Ziadi N. (2011). Sensing of crop nitrogen status: Opportunities, tools, limitations, and supporting information requirements, limitations, and supporting information requirements. HortTechnology.

[15] Kalaji M.H., Dąbrowski P., Cetner M.D., Samborska I.A., Łukasik I., Brestic M., Zivcak M., Horaczek T., Mojski J., Kociel H. (2017). A comparision between different chlorophyll content metres under nutrients deficiency conditions. J. Plant Nut..

[16] Xue L., Cao W., Luo W., Dai T., Zhu Y. (2004). Monitoring leaf nitrogen status in rice with canopy spectral reflectance. Agron. J..

[17] Elwadie M.E., Pierce F.J., Qi J. (2005). Remote sensing of canopy dynamics and biophysical variables estimation of corn in Michigan. Agron. J..

[18] Scotford I.M., Miller P.C.H. (2005). Applications of spectral reflectance techniques in northern European cereal production: A review. Biosyst. Eng..

[19] Zhu Y., Yao X., Tian Y.C., Liu X.J., Cao W.X. (2007). Analysis of common canopy vegetation indices for indicating leaf nitrogen accumulations in wheat and rice. Int. J. Appl. Earth Obs. Geoinf..

[20] Ma B.L., Dwyer L.M., Costa C., Cober E.R., Morrison M.J. (2001). Early prediction of soybean yield from canopy reflectance measurements. Agron. J..

[21] Bronson K.F., Booker J.D., Keeling J.W., Boman R.K., Wheeler T.A., Lascano R.J., Nichols R.L. (2005). Cotton canopy reflectance at landscape scale as affected by nitrogen fertilization. Agron. J..

[22] Gianquinto G., Orsini F., Fecondini M., Mezzetti M., Sambo P., Bona S. (2011). A methodological approach for defining spectral indices for assessing tomato nitrogen status and yield. Eur. J. Agron..

[23] Padilla F.M., Peña-Fleitas M.T., Gallardo M., Thompson R.B. (2015). Threshold values of canopy reflectance indices and chlorophyll meter readings for optimal nitrogen nutrition of tomato. Ann. Appl. Biol..

[24] El-Shikha D.M., Waller P., Hunsaker D., Clarke T., Barnes E. (2007). Ground-basedremote sensing for assessing water and nitrogen status of broccoli. Agric. Water Manag..

[25] Padilla F.M., Peña-Fleitas M.T., Gallardo M., Thompson R.B. (2014). Evaluation of optical sensor measurements of canopy reflectance and of leaf flavonols and chlorophyll contents to assess crop nitrogen status of muskmelon. Eur. J. Agron..

[26] Padilla F.M., Peña-Fleitas M.T., Gallardo M., Thompson R.B. (2017). Determination of sufficiency values of canopy reflectance vegetationindices for maximum growth and yield of cucumber. Eur. J. Agron..

[27] Padilla F.M., de Souza R., Peña-Fleitas M.T., Grasso R., Gallardo M., Thompson R.B. (2019). Influence of time of day on measurement with chlorophyll meters and canopy reflectance sensors of different crop N status. Precis. Agric..

[28] Oliveira L.F., Scharf P.C. (2014). Diurnal variability in reflectance measurements from cotton. Crop Sci..

[29] Cui D., Li M., Zhang Q. (2009). Development of an optical sensor for crop leaf chlorophyll content detection. Comput. Electron. Agric..

[30] Hu M.Q., Mao F., Sun H., Hou Y.Y. (2011). Study of normalized difference vegetation index variation and its correlation with climate factors in the three-river-source region. Int. J. Appl. Earth Obs. Geoinf..

[31] Lu L.Z., Di L.P., Ye Y.M. (2014). A Decision-Tree classifier for extracting transparent plastic-mulched landcover from Landsat-5 TM Images. IEEE J. Sel. Topics Appl. Earth Observ. Remote Sens..

[32] Lu L.Z., Hang D.W., Di L.P. (2015). Threshold model for detecting transparent plastic-mulched landcover using moderate-resolution imaging spectroradiometer time series data: A case study in southern Xinjiang, China. J. Appl. Remote Sens..

[33] Chen Z.X., Wang L.M., Wu W.B., Jiang Z.W., Li H. (2016). Monitoring plastic-mulched farmland by Landsat-8 OLI imagery using spectral and textural features. Remote Sens..

[34] Chen Z.X., Wang L.M., Liu J. (2017). Selecting appropriate spatial scale for mapping plastic-mulched farmland with satellite remote sensing imagery. Remote Sens..

[35] Chen Z.X. (2017). Mapping plastic-mulched farmland with multi-temporal Landsat-8 data. Remote Sens..

[36] Lord D., DesJardins R.L., Dube P.A. (1988). Sun-angle effects on the red and near infrared reflectances of five different crop canopies. Can. J. Remote Sens..

[37] De Souza E.G., Scharf P.C., Sudduth K.A. (2010). Sun position and cloud effects on reflectance and vegetation indices of corn. Agron. J..

[38] Ishihara M., Inoue Y., Ono K., Shimizu M., Matsuura S. (2015). The impact of sunlight conditions on the consistency of vegetation indices in croplands—Effective usage of vegetation indices from continuous ground-based spectral measurements. Remote Sens..

[39] Guan J., Nutter F.W. (2001). Factors that affect the quality and quantity of sunlight reflected from alfalfa canopies. Plant Dis..

[40] Vouillot M.O., Huet P., Boissard P. (1998). Early detection of N deficiency in a wheat crop using physiological and radiometric methods. Agronomie.

[41] Sprent P. (1993). Applied Nonparametric Statistical Methods.

[42] Efron B., Tibshirani R.J. (1993). An Introduction to the Bootstrap.

[43] Sokal R.R., Rohlf F.J. (1995). Biometry: The Principles and Practice of Statistics in Biological Research.

[44] Bartlett J.E., Kotrlik J.W., Higgins C. (2001). Organizational research: Determining appropriate sample size in survey research. Inf. Technol. Learn. Perform. J..

[45] Csillag M., Kertész M., Davidson A., Mitchell S. (2001). On the measurement of diversity-productivity relationships in a northern mixed grass prairie (Grasslands National Park, Saskatchewan, Canada). Community Ecol..

[46] Jongschaap R.E.E., Booij R. (2004). Spectral measurements at different spatial scales in potato: Relating leaf, plant and canopy nitrogen status. Int. J. Appl. Earth Obs. Geoinf..

[47] Jongschaap R.E.E. (2006). Run-time calibration of simulation models by integrating remote sensing estimates of leaf area index and canopy nitrogen. Eur. J. Agron..

[48] McCree K.J. (1972). The action spectrum, absorptance and quantum yield of photosynthesis in crop plants. Agric. Meteorol..

[49] Fitzgerald G.J. (2010). Characterizing vegetation indices derived from active and passive sensors. Int. J. Remote Sens..

[50] Kipp S., Mistele B., Schmidhalter U. (2014). The performance of active spectral reflectance sensors as influenced by measuring distance, device temperature and light intensity. Comput. Electron. Agric..

[51] Solari F., Shanahan J., Ferguson R., Schepers J., Gitelson A. (2008). Active sensor reflectance measurements of corn nitrogen status and yield potential. Agron. J..

[52] Kim Y., Glenn D.M., Park J., Ngugi H.K., Lehman B.L. (2012). Characteristics of active spectral sensor for plant sensing. Trans. ASABE.

[53] Teixeira Crusiol L.G., Corrêa Carvalho J.F., Ribeiro Sibaldelli R.N., Neiverth W., Do Rio A., Ferreira L.C., de Oliveira P.S., Mertz-Henning L.M. (2017). NDVI variation according to the time of measurement, sampling size, positioning of sensor and water regime in different soybean cultivars. Precis. Agric..

[54] CropScan™ (1993). Multi-Spectral Radiometer (MSR): Users Manual and Technical Reference.

[55] Birth G.S., McVey G.R. (1968). Measuring the color of growing turf with a reflectance spectrophotometer. Agron. J..

[56] Ma B.L., Morrison M.J., Dwyer L.M. (1996). Canopy light reflectance and field greenness to assess nitrogen fertilization and yield of maize. Agron. J..

[57] Caturegli L., Gaetani M., Volterrani M., Magni S., Minelli A., Baldi A., Brandani G., Mancini M., Lenzi A., Orlandini S. (2019). Normalized Difference Vegetation Index versus Dark Green Colour Index to estimate nitrogen status on bermudagrass hybrid and tall fescue. Int. J. Remote Sens..

